# Lifetime changes in body fatness and breast density in postmenopausal women: the FEDRA study

**DOI:** 10.1186/s13058-023-01624-5

**Published:** 2023-03-31

**Authors:** Giovanna Masala, Benedetta Bendinelli, Saverio Caini, Giacomo Duroni, Ilaria Ermini, Elisa Pastore, Miriam Fontana, Luigi Facchini, Andrea Querci, Maria Antonietta Gilio, Vincenzo Mazzalupo, Melania Assedi, Daniela Ambrogetti, Domenico Palli

**Affiliations:** 1 Clinical Epidemiology Unit, Institute for Cancer Research, Prevention and Clinical Network (ISPRO), Florence, Italy; 2 Cancer Risk Factors and Lifestyle Epidemiology Unit, Institute for Cancer Research, Prevention and Clinical Network (ISPRO), Via Cosimo Il Veccio 2, 50139 Florence, Italy; 3 Breast Cancer Screening Branch, Institute for Cancer Research, Prevention and Clinical Network (ISPRO), Florence, Italy

## Abstract

**Background:**

High mammographic breast density (MBD) is an established risk factor for breast cancer (BC). Body fatness conveys an increased BC risk in postmenopause but is associated with less dense breasts. Here, we studied the relationship between body fatness and breast composition within the FEDRA (Florence-EPIC Digital mammographic density and breast cancer Risk Assessment) longitudinal study.

**Methods:**

Repeated anthropometric data and MBD parameters (obtained through an automated software on BC screening digital mammograms) were available for all participants, as well as information on other BC risk factors. Multivariate linear regression and functional data analysis were used to longitudinally evaluate the association of body fatness, and changes thereof over time, with dense (DV) and non-dense (NDV) breast volumes and volumetric percent density (VPD).

**Results:**

A total of 5,262 women were included, with anthropometric data available at 20 and 40 years of age, at EPIC baseline (mean 49.0 years), and an average of 9.4 years thereafter. The mean number of mammograms per woman was 3.3 (SD 1.6). Body fatness (and increases thereof) at any age was positively associated with DV and NDV (the association being consistently stronger for the latter), and inversely associated with VPD. For instance, an increase by 1 kg/year between the age of 40 years and EPIC baseline was significantly associated with 1.97% higher DV, 8.85% higher NDV, and 5.82% lower VPD.

**Conclusion:**

Body fatness and its increase from young adulthood until midlife are inversely associated with volumetric percent density, but positively associated with dense and non-dense breast volumes in postmenopausal women.

**Supplementary Information:**

The online version contains supplementary material available at 10.1186/s13058-023-01624-5.

## Introduction

Breast cancer (BC) is the leading cause of cancer morbidity and mortality among women globally, as it accounts for 24.5% of all cancer cases, and 15.5% of all cancer-related deaths [1]. BC risk factors include aspects related to women’s menstrual and reproductive history and use of exogenous sex hormones [2], as well as lifestyle-related characteristics including lack of physical activity and alcohol intake [3]. Concerning anthropometric parameters, body fatness (marked by body mass index (BMI) or waist circumference) is associated with increased BC risk among postmenopausal women but with reduced risk of premenopausal BC, while adult height conveys an increased BC risk regardless of menopausal status [3].

Mammographic breast density (MBD) refers to the proportion of radiologically dense fibroglandular tissue over the total breast, and is usually expressed as percent density. High MBD is an independent risk factor for BC, with consisting evidence that has steadily accumulated in recent years [4–7]. Most BC risk factors are associated with higher MBD, but body fatness stands out as an exception to this rule in postmenopausal women, among whom it is linked to lower MBD while carrying an increased BC risk [3, 8, 9].

Studies on MBD and BC risk were mostly based on qualitative assessment performed by radiologists applying different classification systems and more recently on computer-assisted programs on digitized images. Over the last decade, full-field digital mammograms (FFDMs) have progressively replaced film-screen mammography and automated programs for volumetric MBD assessment has been developed representing an opportunity to obtain standardized repeated measures. So far few studies have investigated the performances of automated quantitative assessment programs in the prediction of BC risk and the associations with BC risk factors [10–12]**.**

In this article, we present the results on the relationship between body fatness and volumetric MBD measures that were obtained within the FEDRA (Florence-EPIC Digital mammographic density and breast cancer Risk Assessment) study based on the Florentine section of the EPIC (European Prospective Investigation into Cancer and nutrition) cohort. In this study, repeated measurements of both anthropometry and MBD, together with detailed information on BC risk factors, were available.

## Methods

### Study population

Between 1993 and 1998, 10,083 clinically healthy women aged 35–64 years residing in the Florence area (Tuscany, Central Italy) were recruited in the EPIC Florence cohort. All study participants signed an informed consent and gave permission to use the data collected during the study. The study was approved by the local Ethics Committee “Azienda Sanitaria Firenze.” All procedures were in accordance with the ethical standards of the institutional and national research committee and with the 1964 Helsinki declaration and its later amendments or comparable ethical standards.

Anthropometric measures were obtained by trained nurses according to an international protocol. Detailed information on reproductive history, smoking and alcohol drinking history, education, physical activity habits, medical history and hormone replacement therapy (HRT) use was collected through a standardized lifestyle questionnaire (LSQ). Dietary information was obtained through a validated Food Frequency Questionnaire (FFQ) [13].

Standardized follow-up procedures have been periodically implemented for the ascertainment of vital status and the identification of cancer cases diagnosed after enrollment.

Information on lifestyle, medical history, reproductive history and anthropometric measures was updated in 2004–2005, after a 9.4 year average follow-up [14]. In 2019, in the frame of the FEDRA (Florence-EPIC Digital mammographic density and breast cancer Risk Assessment) study, an update of the mammographic examinations history of the EPIC female participants was performed through a linkage with the mammographic archives of the local population-based mammographic screening program (performed at ISPRO, in charge of the mammographic screening in the area). For the available FFDMs, quantitative MBD measures were obtained. The FEDRA study was approved by the Ethics Committee of “Area Vasta Centro” of Tuscany Region and participants signed a specific consent form.

The present analysis was focused on 5,262 women, previously enrolled in the Florence-EPIC study, for which follow-up data were available and with at least a FFDM performed after enrollment.

### Breast density measures

The fully automated Volpara_TM_ density software (version 3.1, Matakina Technology, Wellington, New Zealand) was used to obtain total breast volume (cm3), absolute breast dense volume (DV, cm3) and volumetric percent density (VPD, %), from raw (“for processing”) FFDMs data of the retrieved mammograms. The technical characteristics of Volpara system have been already described in details [15]. Briefly, the algorithm computes the thickness of dense tissue at each pixel using the X-ray attenuation of an entirely fatty region as an internal reference. The thickness values over the whole breast region are integrated to obtain the absolute DV. Total breast volume is obtained by multiplying the breast area by the recorded breast thickness, corrected for the breast edge. Non-dense volume (NDV, cm^3^) was derived as the difference between total breast volume and DV. VPD was then obtained from the ratio of DV and total breast volume. In this study, we used the average MBD measures obtained from medio-lateral oblique and cranio-caudal views of the right and left breasts.

### Anthropometric measures

At enrollment in the EPIC study body weight (kg), body height (cm), waist and hip circumferences (cm) were measured by trained nurses according to an international standard protocol [13]. At the 2004–2005 follow-up (9.4 years after enrollment), participants provided self-reported body weight and waist and hip circumference using a meter rule supplied by the study center. Body weight history including weight (kg) at 20 years and at 40 years was also collected.

### Statistical analysis

Body mass index (BMI) was calculated by dividing body weight with squared height (kg/m^2^).

We calculated participants weight changes in three consecutive time intervals: from age 20 to 40; from age 40 to age at enrollment in the EPIC study (after excluding subjects with age at enrollment < 40 years); from age at enrollment in the EPIC study to age at 9.4-year follow-up. The weight changes per year in each interval were calculated. For every time interval, we defined five categories of weight change: stable weight (weight change ± 2 kg); weight gain > 2—≤ 5 kg; weight gain > 5—≤ 10 kg; weight gain > 10 kg; weight loss > 2 kg) [16–18]).

For each participant, we computed the birth index combining the age at every births and their number. Birth index was 0 for nulliparous. A higher birth index indicates a higher number of births occurring at earlier ages [19].

### Multivariable linear regression analysis

Multivariable linear regression models were applied to evaluate the associations of BMI at age 20, at age 40, at age at EPIC enrollment and at 9.4-year follow-up with VPD, DV and NDV at first FFDM. Models were adjusted for: age at mammographic examination, birth index, menopausal status at EPIC enrollment (postmenopausal yes/no) and age at menarche. Similar models, additionally adjusted for BMI at age 20, were applied to evaluate the associations of VPD, DV and NDV with the annual weight change in the three time intervals (from 20 to 40 years, from 40 years to age at EPIC enrollment, from age at EPIC enrollment to age at follow-up) and with the five categories of weight change in every time interval. In all models, VPD, DV and NDV were log-transformed in order to normalize the distribution. Beta coefficients and 95% confidence intervals (CI) were then back transformed (Diff% = (expβ-1) × 100) and presented as percent change in VBD, DV or NDV associated with one unit change in BMI or to one kg of annual weight change or with respect to the reference weight change category. Models further adjusted for family history of breast cancer, and number of years between EPIC enrollment and first FFDM were implemented. Analyses were performed with SAS version 9.2 (SAS Institute, Cary, NC).

### Functional data analysis

Function-On-Scalar (FoS) models [20] adjusted for menopausal status, age at menarche and birth index were implemented to study the effect of BMI at age 20 (Model 1) and at age 40 (Model 2) over a time-varying functions of VPD, DV and NDV (treated as logarithms). A detailed description of the FoS model adopted for this analysis is reported in Additional file 1.

In FoS models, the function of the parameters are estimated all over the considered interval of time, instead of a point estimate given by the classical multiple regression approach. The observed data points are supposed to arise from a function that vary over a determined domain; here, they are functions of VPD, DV and NDV that vary over the subjects’ age. The aim of this approach is to observe the behavior of the MBD measures over time, given a set of scalar covariates (BMI at the ages 20 or 40, menopausal status, age at menarche and birth index). The analysis was conducted using the R “fda” package [21].

The time-dependent analysis requests a high informative dataset with a good representation of the time series. In order to reach a sufficient informative value of the functions, 1,756 women with four consecutive FFDMs and without missing data for the covariates of interest (BMI at the age of 20 or 40, menopausal status, age at the menarche and birth index) were considered for the analysis (Additional file 1: Figure S1)*.* Moreover, given the necessity to observe a common time window to every time series, 390 women who performed the four FFDMs in the age interval 63–68 years were selected (Additional file 1: Figure S2).

For each MBD measure (VPD, DV and NDV), two FoS models were implemented. The estimated functional parameters (*β*(*t*)) described the effect of the BMI at age 20 (Model 1) or the effect of the BMI at age 40 (Model 2) over the time-dependent functions of VPD, DV and NDV in the age interval 63–68. A positive value of *β*(*t*) indicates a positive association between BMI and mammographic density measures and *vice versa*. Confidence intervals for the estimated functional parameters were obtained via bootstrapping, with the number of resampling with replacement being set to 500.

## Results

Selected characteristics of the 5,262 FEDRA study women are reported in Table 1. The mean age at EPIC enrollment was 49.0 years (SD 7.0), the mean age follow-up was 58.9 years (SD 6.8), and the mean age at the first digital mammographic examination was 64.3 years (SD 6.4). The mean number of FFDMs performed by each woman was 3.3 (SD 1.6). Mean BMI was 21.1 kg/m^2^ (SD 2.5) at 20 years, 23.2 kg/m^2^ (SD 3.1) at 40 years, 24.9 kg/m^2^ (SD 4.0) at EPIC enrollment and 25.7 kg/m^2^ (SD 4.3) at follow-up. The mean annual weight change was 0.3 kg/year (SD 0.3) from age 20 to 40, 0.5 kg/year (SD 1.3) from age 40 to EPIC enrollment (after excluding 814 subjects with age at enrollment < 40 years), 0.2 kg/year (SD 0.6) from EPIC enrollment to follow-up. At the first FFDM, the mean VPD was 7.84% (SD 5.10), the mean DV was 47.71 cm^3^ (SD 25.36), and the mean NDV was 701.05 cm^3^ (SD 384.82).

**Table 1:** Main characteristics of the 5,262 FEDRA study women

Characteristics	N ^(a)^	Mean (SD) / %
Age at EPIC enrollment (years)	5262	49.03 (7.02)
Age at follow-up (years)	5262	58.93 (6.82)
Age at first digital mammographic examination (years)	5262	64.29 (6.41)
Volumetric percent density at first digital mammographic examination (%)	5262	7.84 (5.10)
Dense volume at first digital mammographic examination (cm^3^)	5262	47.71 (25.36)
Non-dense volume at first digital mammographic examination (cm^3^)	5262	701.05 (384.82)
Available digital mammographic examinations per woman (*n*)	5262	3.28 (1.63)
Women with four digital mammographic examination (*n*)	2316	44.0%
Age at menarche (years)	5249	12.38 (1.40)
Age at first birth (years)	4457	26.61 (4.34)
Parity (*n*)		
Nulliparous	224	4.8%
One	1614	34.5%
Two	2277	48.6%
Three or more	566	12.1%
Breastfeeding (*n*) (among 4457 women with children)	3917	87.9%
Oral contraceptive (ever use) (*n*)	2791	53.2%
Postmenopause at EPIC enrollment (*n*)	2395	45.6%
Family history of breast cancer (*n*)	370	7.0%
Educational level at EPIC enrollment (*n*)		
None/elementary school	1192	22.8%
Secondary/ professional school	1690	32.3%
High school	1389	26.5%
Degree	965	18.4%
Body height at EPIC enrollment (cm)	5203	160.3 (6.2)
Body weight (kg)		
At age 20 years	4379	54.3 (7.3)
At age 40 years	4492	59.7 (8.6)
At EPIC enrollment	5202	64.0 (10.6)
At follow-up	5252	65.9 (11.4)
Body mass index (kg/m^2^)		
At age 20 years	4336	21.1 (2.5)
At age 40 years	4443	23.2 (3.1)
At EPIC enrollment	5201	24.9 (4.0)
At follow-up	5193	25.7 (4.3)
Annual weight change (kg/years)		
From age 20 to 40	4036	0.3 (0.3)
From age 40 to EPIC enrollment^b^	3707	0.5 (1.3)
From EPIC enrollment to follow-up^c^	5192	0.2 (0.6)
Weight change categories from age 20 to 40 (*n*)		
Weight gain > 10 kg	653	16.2%
Weight gain > 5—≤ 10 kg	1054	26.1%
Weight gain > 2—≤ 5 kg	1184	29.3%
Stable weight (± 2 kg)	885	22.0%
Weight loss > 2 kg	260	6.4%
Weight change categories from age 40 to EPIC enrollment (*n*)		
Weight gain > 10 kg	549	14.8%
Weight gain > 5—≤ 10 kg	900	24.3%
Weight gain > 2—≤ 5 kg	956	25.8%
Stable weight (± 2 kg)	1055	28.4%
Weight loss > 2 kg	247	6.7%
Weight change categories from EPIC enrollment to follow-up (*n*)		
Weight gain > 10 kg	296	5.7%
Weight gain > 5—≤ 10 kg	844	16.3%
Weight gain > 2—≤ 5 kg	1181	22.8%
Stable weight (± 2 kg)	1931	37.1%
Weight loss > 2 kg	940	18.1%

^a^ Because of some missing values not all reach the total number of participants

^b^ A total of 814 women enrolled before age 40 have been excluded. Mean 11.1 (SD 5.3) years between age 40 and EPIC enrollment

^c^ Mean 9.4 (SD 1.1) years between EPIC enrollment and follow-up

### Multivariable linear regression analysis

Overall, as reported in Table 2, BMI was inversely associated with VPD and positively with DV and NDV: 1 kg/m^2^ increase in BMI at age 20 was associated with a 4.42% lower VPD, a 0.93% higher DV and a 5.98% higher NDV; a 6.59% lower VPD, a 2.20% higher DV and a 10.07% higher NDV were shown for the same increase in BMI at age 40; for each 1 kg/m^2^ increase in BMI at EPIC enrollment, we observed a 6.23% lower VPD, a 1.95% higher DV and a 9.34% higher NDV; a 6.32% lower VPD, a 1.98% higher DV and a 9.51% higher NDV were shown for increased BMI at follow-up (all *p*-values < 0.001).

**Table 2:** Multivariable-adjusted regressions between adiposity measures, weight changes and mammographic density at first digital mammographic examination among 5262 women in the FEDRA study (VPD = Volumetric percent density; DV = dense volume; BMI = body mass index; 95% CI = 95% confidence interval) ^a^

	VPD (%)		DV (cm^3^)		NDV (cm^3^)	
	%Diff (95% CI)^b^	*p*-value	%Diff (95% CI)^b^	*p*-value	%Diff (95% CI)^b^	*p*-value
BMI at age 20 years (kg/m2)	− 4.42 (− 5.05 to − 3.78)	< 0.0001	0.93 (0.37 to 1.50)	0.001	5.98 (5.24 to 6.71)	< 0.0001
BMI at age 40 years (kg/m2)	− 6.59 (− 7.07 to − 6.11)	< 0.0001	2.20 (1.74 to 2.67)	< 0.0001	10.07 (9.52 to 10.63)	< 0.0001
BMI at EPIC enrollment (kg/m2)	− 6.23 (− 6.57 to − 5.89)	< 0.0001	1.95 (1.61 to 2.29)	< 0.0001	9.34 (8.95 to 9.73)	< 0.0001
BMI at follow-up (kg/m2)	− 6.32 (− 6.62 to − 6.02)	< 0.0001	1.98 (1.68 to 2.29)	< 0.0001	9.51 (9.18 to 9.84)	< 0.0001
Annual weight change from age 20 to age 40 (kg/year)^c^	− 44.91 (− 47.76 to − 41.90)	< 0.0001	23.61 (17.88 to 29.62)	< 0.0001	136.79 (124.79 to 149.44)	< 0.0001
Annual weight change from age 40 to EPIC enrollment (kg/year)^c^	− 5.82 (− 7.17 to − 4.45)	< 0.0001	1.97 (0.72 to 3.24)	0.002	8.85 (7.26 to 10.46)	< 0.0001
Annual weight change from EPIC enrollment to follow-up (kg/year)^c^	-17.64 (− 19.99 to − 15.21)	< 0.0001	5.99 (3.37 to 8.68)	< 0.0001	31.24 (27.39 to 35.20)	< 0.0001
Weight change from age 20 to age 40 (kg)^c^						
Weight gain > 10 kg	− 37.71 (− 40.93 to − 34.32)	< 0.0001	19.74 (14.20 to 25.54)	< 0.0001	100.83 (90.81 to 111.38)	< 0.0001
Weight gain > 5—≤ 10 kg	− 31.87 (− 35.00 to − 28.58)	< 0.0001	14.00 (9.30 to 18.90)	< 0.0001	73.75 (66.01 to 81.85)	< 0.0001
Weight gain > 2—≤ 5 kg	− 19.64 (− 23.22 to − 15.89)	< 0.0001	5.42 (1.21 to 9.81)	0.01	34.38 (28.58 to 40.44)	< 0.0001
Stable weight (± 2 kg)	1.00 (ref.)		1.00 (ref.)		1.00 (ref.)	
Weight loss > 2 kg	19.97 (11.40 to 29.20)	< 0.0001	-7.13 (-13.08 to -0.76)	0.03	-24.04 (-29.30 to -18.40)	< 0.0001
Weight change from age 40 to EPIC enrollment (kg)^c^						
Weight gain > 10 kg	− 35.91 (− 39.48 to − 32.12)	< 0.0001	13.97 (8.37 to 19.86)	< 0.0001	84.68 (74.45 to 95.51)	< 0.0001
Weight gain > 5—≤ 10 kg	− 25.80 (− 29.34 to − 22.08)	< 0.0001	6.12 (1.66 to 10.78)	0.007	46.95 (40.00 to 54.27)	< 0.0001
Weight gain > 2—≤ 5 kg	− 13.59 (− 17.64 to 9.34)	< 0.0001	5.70 (1.33 to 10.26)	0.01	23.80 (18.04 to 29.85)	< 0.0001
Stable weight (± 2 kg)	1.00 (ref.)		1.00 (ref.)		1.00 (ref.)	
Weight loss > 2 kg	− 4.74 (− 11.77 to 2.84)	0.21	2.14 (− 4.52 to 9.25)	0.54	7.36 (− 0.51 to 15.86)	0.07
Weight change from EPIC enrollment to follow-up (kg)^c^						
Weight gain > 10 kg	− 33.96 (− 38.47 to − 29.11)	< 0.0001	11.73 (5.09 to 18.79)	0.0004	75.61 (63.40 to 88.74)	< 0.0001
Weight gain > 5—≤ 10 kg	− 21.56 (− 25.19 to − 17.76)	< 0.0001	10.25 (5.82 to 14.86)	< 0.0001	43.99 (37.21 to 51.10)	< 0.0001
Weight gain > 2—≤ 5 kg	− 8.45 (− 12.26 to 4.47)	< 0.0001	5.00 (1.21 to 8.93)	0.01	15.73 (10.83 to 20.84)	< 0.0001
Stable weight (± 2 kg)	1.00 (ref.)		1.00 (ref.)		1.00 (ref.)	
Weight loss > 2 kg	1.86 (− 2.76 to 6.70)	0.44	− 0.58 (− 4.50 to 3.49)	0.78	-2.82 (− 7.30 to 1.)	0.24

^a^ All models were adjusted for age at mammographic examination, birth index, postmenopausal status *(enrollment, yes/no),* age at menarche

^b^ The %Diff represents the percent change of VPD or DV or NDV associated to one unit change in BMI or 1 kg/year of weight gain or with respect to the stable weight group

^c^ Model additionally adjusted for BMI at age 20

Overall, annual weight change at the considered age intervals was inversely associated with VPD and positively associated with DV and NDV: 1 kg/year of annual weight change between age 20 and age 40 was associated with a 44.91% lower VPD, a 23.61% higher DV and a 136.79% higher NDV; 1 kg/year of annual weight change between age 40 and age at EPIC enrollment was associated with a 5.82% lower VPD, a 1.97% higher DV (all p-values 0.002) and an 8.85% higher NDV; 1 kg/year of annual weight change between age at EPIC enrollment and age at follow-up was associated with a 17.44% lower VPD, a 5.99% higher DV and a 31.24% higher NDV (all p-values < 0.0001). In the considered age intervals, weight gain categories (2–5 kg, 5–10 kg, > 10 kg) were increasingly associated with a significantly lower VPD and to significantly higher DV and NDV compared to stable weight (Table 2).

Further adjustments for HRT use, family history of BC and years between EPIC enrollment and FFDM did not substantially changed the results.

### Functional data analysis

**Fig. 1:**
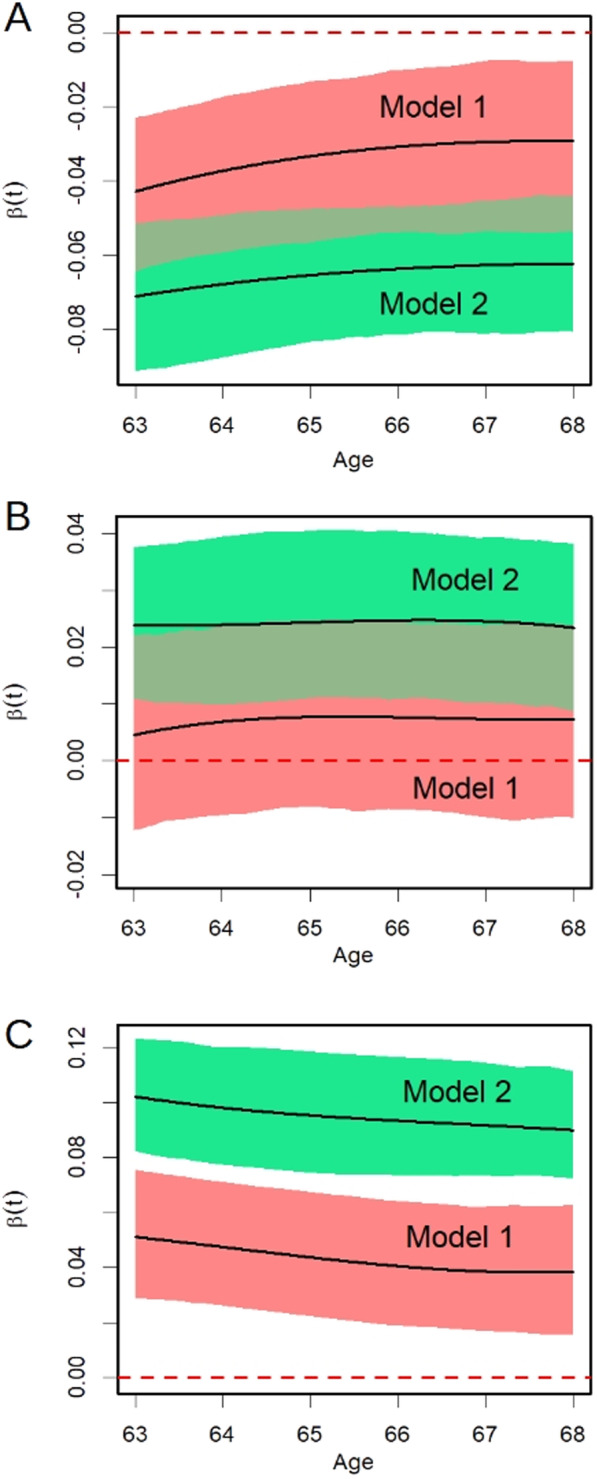
Function-on-Scalar models estimating, among 390 women from the FEDRA study in the age interval 63–68, the effect (*β*(t)) of the body mass index (BMI, kg/m^2^) over a time-varying function of: **A** the mammographic volumetric percent density; **B** the breast dense volume; **C** the breast non-dense volume. In Model 1 the BMI at age 20 was considered. In Model 2, the BMI at age 40 was considered. A positive value of *β*(*t*) indicates a positive association between BMI and mammographic density measures and vice versa. All the models were adjusted for menopausal status, age at menarche and birth index. The 95% confidence intervals were obtained through 500 bootstrap sampling

The estimated functional parameters (*β*(*t*)), describing the effect of the BMI at age 20 (Model 1) and age 40 (Model 2) over the time-dependent functions of VPD, DV and NDV in 390 women in the age interval 63–68, are reported in Fig. 1 together with 95% confidence bands. In both models, a negative association between BMI and VPD (Fig. 1A) and a direct association with DV emerge (Fig. 1B). An increase in BMI (whether at the age of 20 or 40) was also associated with an increase in NDV (Fig. 1C). For all the MBD measures, the absolute values of the β(t) are always larger in Model 2, referred to the BMI at age 40, in respect to Model 1.

## Discussion

We analyzed data from over 5,000 women participating in a large population-based prospective cohort with repeated anthropometric and volumetric mammographic breast density measures. We found that higher BMI at specific point in one’s life (and increases thereof) are inversely associated with volumetric percent density and positively associated with absolute dense volume and non-dense volume in postmenopausal women. Consistent findings emerged in two different analytical approaches, one of which (multivariate linear regression analysis) investigated the prospective association between BMI from young adulthood to late midlife and MBD measures taken at the first available FFDM, while the other (functional data analysis) studied the relationship between BMI, measured at age 20 or 40, and VPD, DV and NDV obtained by repeated FFDMs between 63 and 68 years of age.

Previous cross-sectional studies investigating the association between body fatness and volumetric measures of breast density, recently reviewed by Soguel et al., showed an inverse association of BMI with VPD and a positive association of BMI with DV and NDA [9]. More recently, a study carried out in 383 premenopausal women participating to mammographic routine screening showed that adiposity measures and weight change during adulthood were inversely associated with VPD, and positively associated with DV and NDV [22]. Another large study of 24,840 women (81% postmenopausal) found that BMI at age 18 (late adolescent BMI) was inversely associated with Cumulus measures of VPD on processed FFDM images [23]. Finally, a study in 367 postmenopausal women undergoing annual screening mammogram aimed to evaluate the associations of BMI changes from ages 18 and 30 to age at mammogram with volumetric measures of breast density, assessed using Volpara, found that BMI increase over the life course was inversely associated with VPD, positively associated with NDV and, more weakly, with DV [24].

Notably in our large longitudinal study, BMI at different ages was positively associated with both radiologically dense (e.g., fibroglandular tissue) and non-dense (e.g., fat) breast volumes, being the magnitude of the association stronger for the latter. Therefore, the inverse association between measures of overweight and obesity (such as BMI) and VPD, seems primarily due to the accumulation in the breast of non-dense fatty tissue although an effect on fibroglandular tissue is also evident. Moreover, our results based on functional data analysis suggest that higher BMI in pre-menopause (namely at 20 and 40 years of age) is associated with increased DV and NDV over a range of age (63–68 years) where BC incidence rates are considerably higher. As showed by the International Agency for Research on Cancer in the 2017 volume "Cancer Incidence in Five Continents," the greater age-specific incidence for female breast cancer was after age 60 [25]. In particular, in the 2020 in-depth study from Saika K et al., it was observed a peak of incidence rate at age 65 in Italy [26]. Understanding the relationship between BMI at an earlier age and mammography density in this age interval is therefore of particular interest.

MBD has been considered as a possible intermediate endpoint in randomized trials aimed to evaluate the efficacy of specific interventions aimed to reduce breast cancer risk. In a previous study from Cuzick et al. (case–control study nested in the IBIS-I chemoprevention trial of tamoxifen vs placebo), a 63% reduction in breast cancer risk was observed in women on tamoxifen who experienced a 10% or greater reduction in percent breast density [27]. Studies on breast cancer patients treated with endocrine therapies [28–30] suggested an improved breast cancer survival and lower recurrence rates for MBD reductions ranging from approximately 10% to more than 20%. In our study, a reduction in VPD ranging from 5.8% to 44.9% associated with weight changes in different period of life was observed. Although, unlike our study, all the studies previously mentioned used two-dimensional MBD measures based on film mammograms and obtained on different scales, and therefore comparisons are difficult, and a clinically relevant role of MBD changes showed with our study could be hypothesized.

Mammographic breast density and BMI are related to each other, and are independent risk factors for breast cancer. As showed in other studies, we confirm that higher BMI in all the considered time point in postmenopausal women's life is inversely associated with volumetric percent density and directly associated with dense and non-dense breast volume, with a greater increase in non-dense adipose tissue than in dense fibroglandular tissue. Because of this discrepancy, body size and mammographic density are confounders of one another in their association with risk of breast cancer. Failure to take this relationship into account leads to the underestimation of the effects of these risk factors both on pre- and postmenopausal women [31, 32].

The biological mechanisms by which overweight/adiposity increases breast dense tissues, in addition to the non-dense tissues, are still to be fully elucidated. Of note, breast adipose tissue is well-known to work as an endocrine organ by secreting adipokines, growth factors, pro-inflammatory cytokines, aromatase (the key enzyme in the conversion of androgens into estrogens, which stimulate epithelial proliferation of the mammary gland) and other factors that regulate cellular processes in epithelial cells. Breast adipose tissue is part of the microenvironment surrounding the fibroglandular zone of the breast, thus the complex crosstalk between adipose tissue and breast epithelial cells may be hypothesized to be a key mechanism by which overweight and obesity affect dense fibroglandular tissue and breast cancer risk [9, 33]. An association between blood levels of breast mitogens and mammographic density in postmenopausal women was documented and suggested to be a biological basis for the association with BC risk [34].

Our study has several strengths. The assessment of volumetric MBD measures from digital mammograms through an automated and validated software ensured the reproducibility of measures and the possibility of comparison with other published studies in the same field. The FEDRA study is nested in an ongoing general population-based prospective cohort that was periodically linked with the mammogram archives of the local breast cancer screening program, which ensures representativeness and generalizability of its results. The present investigation is longitudinal in nature as it takes advantage of repeated measurements of both the exposure (anthropometric measures), the response variable (MBD measurements), and potential confounders. The availability of information on both the exposure and the outcome at multiple points over the course of participants’ lifetime allowed to apply “functional data analysis,” a highly innovative analytical approach that compute functions of variable(s) of interest that vary over a given domain and that nicely apply to longitudinal studies. On the other hand, functional data analysis is more demanding in terms of data availability, and this is the reason why it could be run only in a subset of the study population that fully met the requirements of the method.

In conclusion, we found that body fatness and its increase across a woman’s lifespan (from young adulthood until midlife) are inversely associated with volumetric percent density, but positively associated with dense breast volumes (e.g., fibroglandular tissue) and non-dense volume in postmenopausal women. Overall, our findings will help gain a better understanding of the complex relationship linking overweight/obesity, breast composition, and the risk of postmenopausal BC. These results may have implications for BC prevention adding evidence backing the recommendation to keep weight under control over the course of life.

## Supplementary Information

**Additional file 1**. Supplementary Methods, Figures S1 and S2, and References.

## Data Availability

The datasets generated during and/or analyzed during the current study are not publicly available due to participants privacy protection but are available from the corresponding author on reasonable request.

## Abbreviations

BC: Breast cancer
BMI: Body mass index
DV: Dense breast volume
EPIC: European prospective investigation into cancer and nutrition
FEDRA: Florence-EPIC digital mammographic density and breast cancer risk assessment
FFDM: Full-field digital mammogram
FFQ: Food frequency questionnaire
FoS: Function-on-scalar
HRT: Hormone replacement therapy
LSQ: Lifestyle questionnaire
MBD: Mammographic breast density
NDV: Non-dense breast volume
VPD: Volumetric percent density
