# Supplementary material for: Lifetime changes in body fatness and breast density in postmenopausal women: the FEDRA study
Source: Breast Cancer Res. 2023 Mar 31;25:35. doi: 10.1186/s13058-023-01624-5 (PMC10067176; doi:10.1186/s13058-023-01624-5)
Supplement: Supplementary file 1 — Additional file 1. Supplementary Methods, Figures S1 and S2, and References. [file 13058_2023_1624_MOESM1_ESM.pdf]

Additional File 1

Functional data analysis methods

In the latest decades, thanks to increasing capacity in computer calculus and data storing, it become possible to approach longitudinal studies through functional data analysis, that represents observed data as functions of variable(s) of interest that vary over a given domain.
In this work we applied the Function-On-Scalar (FoS) model to study the variability of mammographic breast density over time.
The FEDRA study includes 5,262 women with a mean number of 3.28 (SD 1.63) consecutive digital mammographic examination for each subject. We studied the effect of covariates of interest, with a main focus on the body mass index (BMI) measured at the ages 20 and 40, over a time-varying function of the mammographic density.
The FoS model proposed below and the notation that follows have been deeply discussed by Ramsay J.O. and Silverman B.W. [1]:

$$y_i(t)=\beta_0(t)+\sum_{j=1}^{q} x_{ij}\beta_j(t)+\epsilon_i(t).$$

Where $y_i(t)$ is the dependent variable for which a prediction for its expected value is obtained through the linear predictor $X\beta$. The main differences between the classical linear approach and the functional data analysis is that the observed data points, whether they are related to describe the dependent or independent variables, are supposed to arise from a function that vary over a determined domain, here time (or the patients' age).
The functional arguments are the dependent variable (here noted as a function of time for the *i*-th individual), and the β coefficients that describe the effect of the covariates over time. The ϵ_i term is the error term which is supposed to arise from a stochastic process with an expected value equal to 0 [2].

Such functions are obtained consequently to a basis expansion; in fact, functional data are described through a linear combination of what are usually called functional building blocks. More generally, an x function of t is specified by K basis functions and the relative c_k coefficients:

$$x(t)=\sum_{k=1}^{K} c_k\phi_k(t).$$

Lots are the choices to construct the basis system. In this analysis we chose b-splines (splines builded through a set of basis functions which are themselves splines) a flexible tool to deal with non-periodic functions (such are supposed to be the functions analysed). A more in-depth description can be found in James et al. [3].

The time dependent analysis requests a high informative dataset with a good representation of the time series. In our analysis, in order to reach a sufficient informative value of the functions and, in the same time, to maintain enough individuals in the analyses 1,765 subjects were considered, with 4 consecutive mammographic examination and no missing data for the following study covariates: BMI at the age of 20, BMI at the age of 40, menopausal status, age at the menarche and birth index.

The analysis has been developed by R using the fda package [4].
The 4 consecutive mammographic examinations were unevenly spaced, and the observation windows varied through individuals, such that the t_0 (age at first mammographic examination) for the subject *i* could be > t_n (age at last mammographic examination) for the subject *j*. A direct use of fda package was not possible under these conditions. To overcome this issue, for each subject, we used order 4 b-spline basis to extrapolate, for a given time sequence, the unobserved values common to each time-series.
Moreover, given the necessity to observe a time window common for every time series, the age interval 63-68 was studied to be the trade-off between the width of the window (or the information carried by each time series) and the number of subjects (or time series) in the study. A total of 390 women met these requirements (Supplementary figure 1).
For the 390 selected women, cubic splines, defined in the age interval 63-68, have been used to specify the functions required by the fda package for the following mammographic density parameters (dependent variables) treated as logarithm: volumetric percent density (VPD); absolute dense volume (DV, cm^3); absolute non dense volume (NDV, cm^3). Cubic splines for the VPD are reported on Supplementary figure 2.
The obtained curves served the purpose to estimate FoS models that explain the effect of the BMI ($\beta(t)$) on VPD, DV, and NDV over time, respectively. Two FoS models are proposed for each dependent variable according to the value of the BMI measured at age 20 (Model 1) and at age 40 (Model 2).
An analytical evaluation of the confidence intervals of the estimated $\beta(t)$ was not feasible given the nature of the data. To overcome this issue we applied the bootstrap technique [3] with 500 resampling.

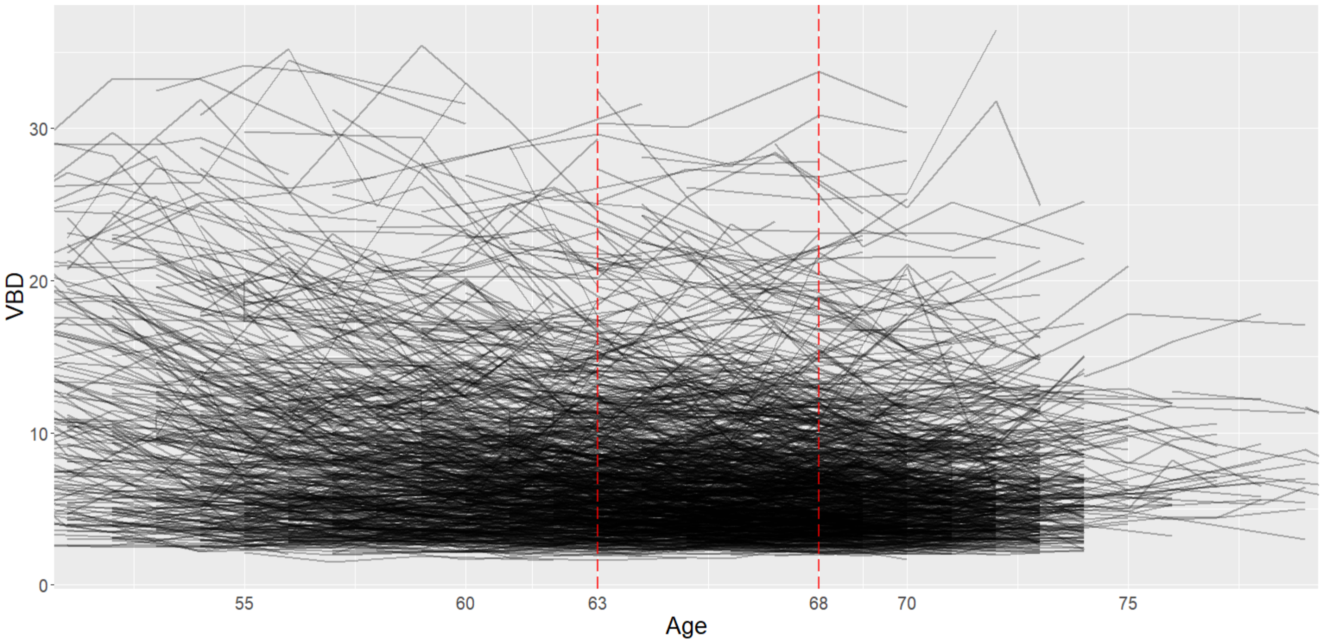

Su**pplementary figure 1:** Time series and the selected age interval (dashed lines) in the 1765 women from the FEDRA study. For each woman the time series of the volumetric percent density (VPD) values obtained from the 4 consecutive mammographic examinations is reported. A total of 390 women performed 4 consecutive mammographic examination in the selected age interval 63-68.

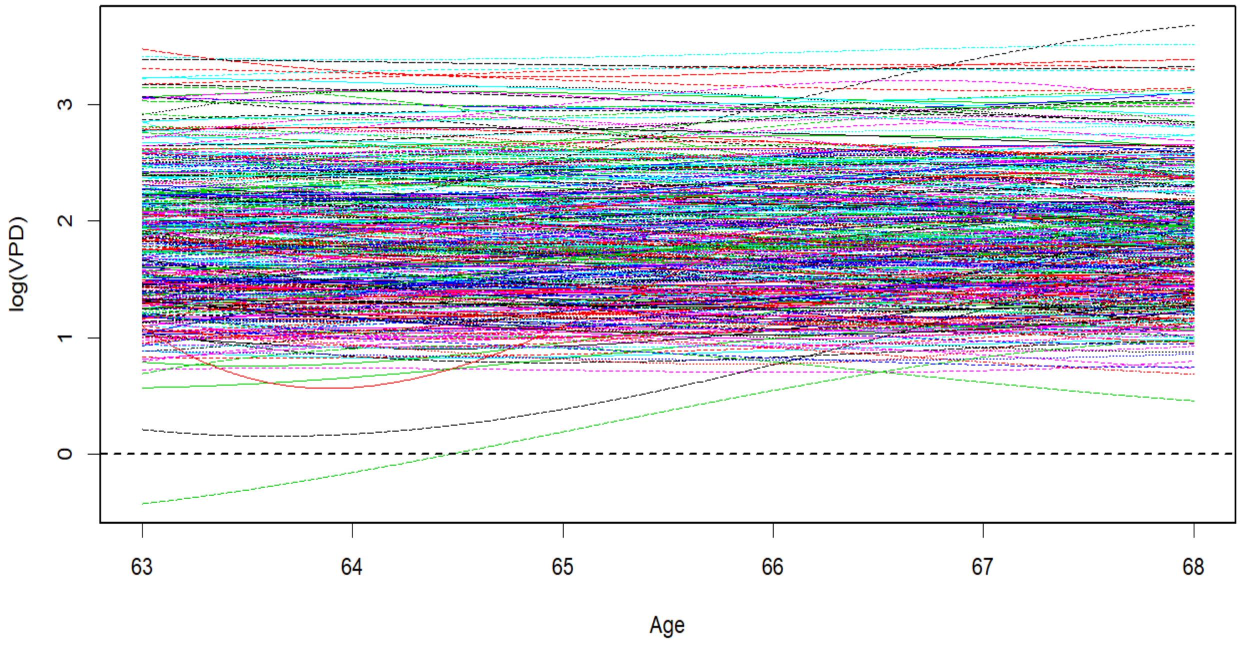

Supplementary figure 2: Cubic splines for the volumetric percent density (VPD), treated as logarithm among the 390 women from the FEDRA study selected for the functional data analysis.

References

1. Ramsay JO, Silverman BW. Functional Data Analysis. 2nd ed. New York, NY: Springer; 2005.

2. Reiss PT, Huang L, Mennes M. Fast function-on-scalar regression with penalized basis expansions. Int J Biostat. 2010;6(1):Article 28. doi: 10.2202/1557-4679.1246. PMID: 21969982.

3. James G, Witten D, Hastie T, Tibshirani R. An Introduction to Statistical Learning. 1st ed. New York, NY: Springer; 2013.

4. Ramsay JO, Hooker G, Graves S, Functional data analysis with R and MATHLAB. 2009 ed. New York, NY: Springer; 2009.
